# Exposure of kale root to NaCl and Na_2_SeO_3_ increases isothiocyanate levels and Nrf2 signalling without reducing plant root growth

**DOI:** 10.1038/s41598-018-22411-9

**Published:** 2018-03-05

**Authors:** Sun Young Kim, Jai-Eok Park, Eun Ok Kim, Sue Ji Lim, Eui Jeong Nam, Ji Ho Yun, GyHye Yoo, Sang-Rok Oh, Hyoung Seok Kim, Chu Won Nho

**Affiliations:** Convergence Research Center for Smart Farm Solution, Korea Institute of Science and Technology (KIST), Gangneung Institute of Natural Products, Gangneung, Gangwon-do, 25451 Korea

## Abstract

A plant factory is a closed cultivation system that provides a consistent and modified environment for plant growth. We speculated that treatment of kale (*Brassica oleracea*) grown in a plant factory with NaCl, Na_2_SeO_3_, or both would increase the bioactive phytochemical levels including glucosinolates (GLSs) and isothiocyanates (ITCs), the key molecules in cancer prevention. The kale was harvested and analysed after treatment with NaCl and Na_2_SeO_3_ alone or in combination for 1 or 2 weeks. Exposure to NaCl alone but not Na_2_SeO_3_ increased plant root growth. Levels of sinigrin were increased by a 2-week exposure to Na_2_SeO_3_ alone or in combination with NaCl, whereas no changes were observed in glucoraphanin and gluconasturtiin gluconasturtiin levels. Importantly, the ITC concentration was affected by 2-week treatment with both compounds. To evaluate the bioactivity of kale, HepG2 human hepatoma cells were treated with plant extract for 6 h. Only the extract of kale roots exposed to a combination NaCl and Na_2_SeO_3_ for 2 weeks showed an increased expression of nuclear factor erythroid 2-related factor (Nrf2), which regulates genes encoding antioxidant proteins. These data suggest that co-treatment with NaCl and Na_2_SeO_3_ increased the ITC content and chemopreventive effects of kale root.

## Introduction

In plants grown in an open field, the yield and quality are dependent on environmental conditions such as temperature, the amount of solar radiation, and the photoperiod^1^. However, in a plant factory with controlled artificial lighting, temperature, CO_2_ concentration, and airflow, specific conditions can be maintained to produce plants with consistent quality^2^. In hydroponic cultures, plants are grown without soil in mineral nutrient solutions^3^. This has several advantages over soil cultures including flexibility in the control of the root-zone environment and mechanisation of cultivation processes^4^. Thus, plants can be exposed to a variety of artificial stimuli and physiological stresses to maximise the yield of the target material^3^.

Glucosinolates (GLSs) are sulphur-containing secondary metabolites synthesised by various plants of the Brassiceae family (*Brassica* genus)^5^. The intake of GLS-containing vegetables has nutritional benefits that are attributable not only to the molecules but also their metabolites. GLSs are hydrolysed by plant myrosinase during cooking or by bacterial myrosinase during digestion and are further broken down into products such as isothiocyanates (ITCs), nitriles, oxazolidin-2-thiones, and indole-3-carbinols depending on the pH, temperature, and activity status of myrosinase^6^. GLS metabolites have chemopreventive properties^7^; ITCs including sulphoraphane (SFN)^8^, phenylethyl ITC (PEITC)^9^, and allyl ITC(AITC)^10^ are promising therapeutic candidates for cancer treatment^11^ that induce the expression of phase II detoxification enzymes (e.g. haeme oxygenase-1 and NAD(P)H: quinone oxidoreductase 1) via nuclear factor erythroid 2-related factor (Nrf)2^12^. The chemopreventive effects of some plants can be improved by modifying the plant growth conditions, for instance by supplementing S^13^ or Selenite (Se)^14^ or increasing CO_2_ or NaCl concentrations^15^, which in turn affect GLS levels. However, it is not known whether manipulating specific components can enhance ITC levels in plants, which could have important implications for cancer prevention.

Therefore, the present study investigated whether endogenous GLS or ITC contents can be increased in kale plants (*Brassica oleracea* L. var. sabellica) cultivated in a closed plant growth system (i.e. plant factory). We hypothesized that growing kale under stressful conditions would increase the concentrations of three desirable GLSs (glucoraphanin, gluconasturtiingluconasturtiin, and sinigrin) and ITCs (SFN, PEITC, and AITC), which would enhance the chemopreventive potential of the plants. To this end, kale plants were exposed to NaCl, Na_2_SeO_3_ (selenite), or both and the GLS and ITC contents were measured. We also examined target protein expression in HepG2 cell lines treated with the extract of kale roots or shoots grown under stressful experimental conditions.

## Results

### Kale shoot but not root growth is affected by NaCl and Na_2_SeO_3_ levels

**Figure 1:**
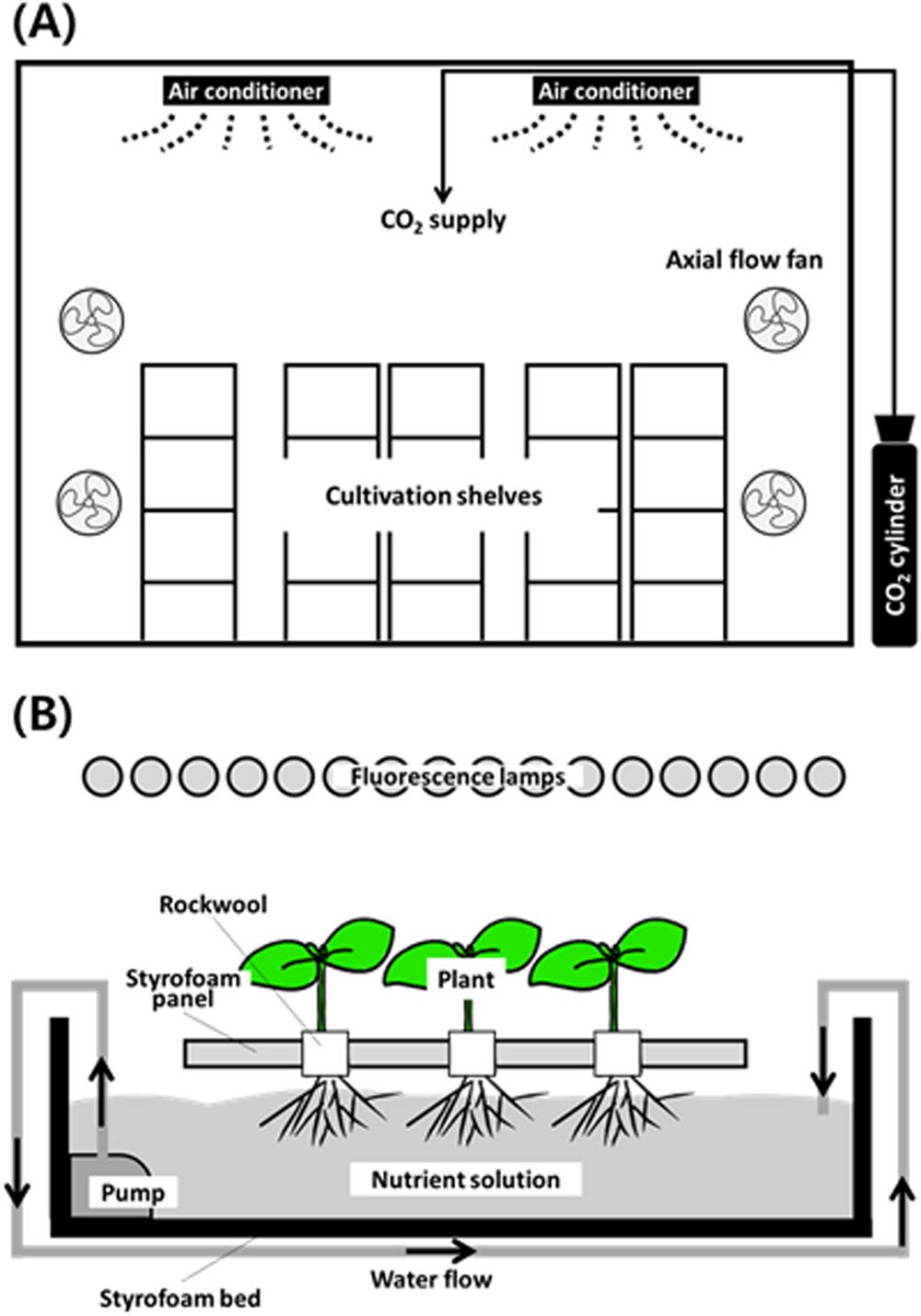
Plant factory. Schematic illustration of (**A**) cultivation room and (**B**) cultivation system. Nutrient solution was circulated using an aquatic pump.

**Figure 2:**
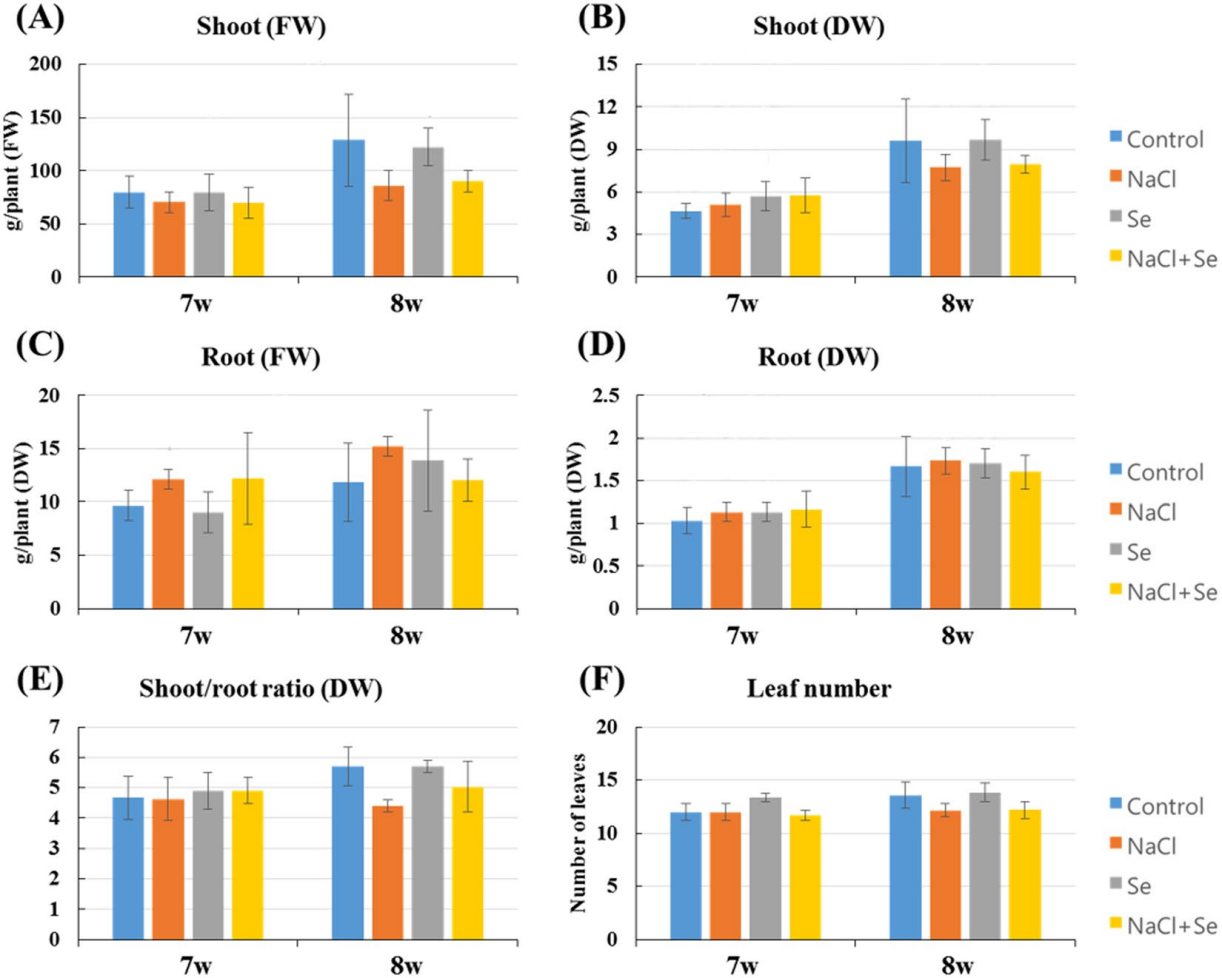
Growth rate of kale plants under different stress condition. Plants were grown in the plant factory and were untreated (control, blue) or treated with sodium chloride (NaCl, 80 Mm; orange), selenite (Na_2_SeO_3_, 2 mg∙l^−1^: Se, gray), or both (NaCl + Se, yellow) for 6 weeks. Plants were harvested at week 7 or 8, and different parts were analysed. Fresh (**A** and **C**) and dry (**B** and **D**) weights of shoots and roots were determined. We also analysed the (**E**) shoot/root ratio and (**F**) leaf number. Statistical comparisons of significance are shown in adjoining lines.

We investigated the effect of NaCl and Na_2_SeO_3_ alone or in combination in nutrient solution on the growth rate of kale cultivated for 1 or 2 weeks in a plant factory system (Fig. 1). We observed that there was no significant differences between control and treated condition at week 7 and 8 (Fig. 2A,B,C and D). In the shoot/root ratio, we also observed that there was no differences between the control and treatments at week 7 and 8, suggesting that neither NaCl nor Na_2_SeO_3_ influenced on growth of kale (Fig. 2E and F). We compared the effect of NaCl and Na_2_SeO_3_ alone or combined between the controls and treated plants only from week 8, which was the experimental end point (Table 1). The results of the comparison of the biomass between the control and treatments at week 8 indicated significant differences in the shoot/root ratio, which was lower in the NaCl-treated plants than in those not treated with NaCl nutrient solution. NaCl treatment alone had significant effects on the shoot fresh weight, shoot/root ratio, and leaf number (Table 1). Thus, NaCl stress did not reduce kale root growth but had a negative effect on the shoots.

**Table 1:** Fresh and dry weight of shoot and root (g/plant), shoot/root dry weight ratio, and leaf number of kale plants grown under control (no NaCl or selenite), NaCl (80 mM), selenite (2 mg/L Na_2_SeO_3_) and combination (80 mM NaCl and 2 mg/L Na_2_SeO_3_) treatments at week 8.

NaCl (mM)	Na_2_SeO_3_ (mg∙L^−1^)	Shoot (FW)	Root (FW)	Shoot (DW)	Root (DW)	Shoot/root ratio (DW)	Leaf number
0	0	128.93 ± 43.42^a,Y^	11.83 ± 3.69^a^	9.63 ± 2.95^a^	1.67 ± 0.35^a^	5.70 ± 0.62^b^	13.57 ± 1.25^a^
0	2	122.30 ± 17.85^a^	13.90 ± 4.73^a^	9.67 ± 1.42^a^	1.70 ± 0.17^a^	5.70 ± 0.20^b^	13.83 ± 0.90^a^
80	0	85.97 ± 14.18^a^	15.20 ± 0.95^a^	7.73 ± 0.93^a^	1.73 ± 0.15^a^	4.40 ± 0.20^a^	12.17 ± 0.64^a^
80	2	90.10 ± 9.92^a^	12.00 ± 2.00^a^	7.93 ± 0.64^a^	1.60 ± 0.20^a^	5.03 ± 0.84^ab^	12.20 ± 0.78^a^
NaCl		*^Z^	NS	NS	NS	*	*
Na_2_SeO_3_		NS	NS	NS	NS	NS	NS
NaCl × Na2SeO3		NS	NS	NS	NS	NS	NS

^Y^Different letters indicate statistical differences in mean within each column (Duncan, *P* < 0.05, n = 3 for each treatment). ^Z^Means ± standard deviation (SD, n = 3) significance at P < 0.05 according to analysis of variance (ANOVA) test, not significant at P > 0.05 (NS) and significant at *P < 0.05.

### Comparison of GLS contents of kale shoots cultivated in plant factory vs. open field

**Figure 3:**
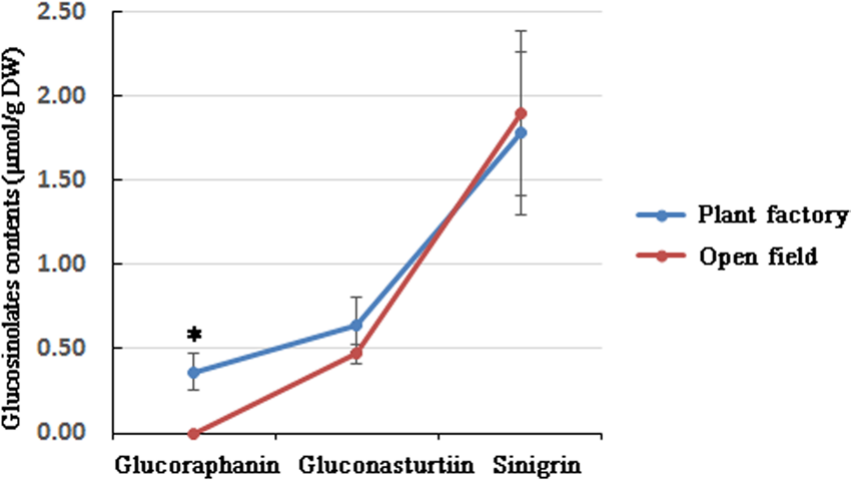
High-performance liquid chromatography (HPLC) analysis of levels of three glucosinolates (GLSs) in kale shoots. Plants were grown in the plant factory or an open field. Shoots were harvested, freeze-dried, 0.2 g of the powder was extracted and then analysed for glucoraphanin, gluconasturtiin, and sinigrin content using HPLC. Expression levels were detected in three separate samples. Statistical comparisons of significance are shown in adjoining lines. *P < 0.05.

The GLSs glucoraphanin, gluconasturtiin, and sinigrin are bioactive molecules that are effective as metabolite precursors^8,9^ or by themselves^16^. To determine whether controlling the conditions of growing plants affected GLS production, we compared the amounts of glucoraphanin, gluconasturtiin, and sinigrin produced by plants grown in the plant factory and the open field. Higher levels of glucoraphanin and gluconasturtiin were detected in kale shoots from the plant factory than in plants from the open field (0.36 ± 0.11 vs. 0.00 and 0.64 ± 0.16 vs. 0.47 ± 0.06, respectively), and the difference of glucoraphanin was statistically significant whereas gluconasturtiin was not. In contrast, sinigrin levels were comparable between plants grown in both environments (1.78 ± 0.48 vs. 1.90 ± 0.49 (Fig. 3)). These results indicate that the plant factory environment enhanced the glucoraphanin and gluconasturtiin production in kale shoots.

### Effects of NaCl and Na_2_SeO_3_ on GLS and ITC contents

**Figure 4:**
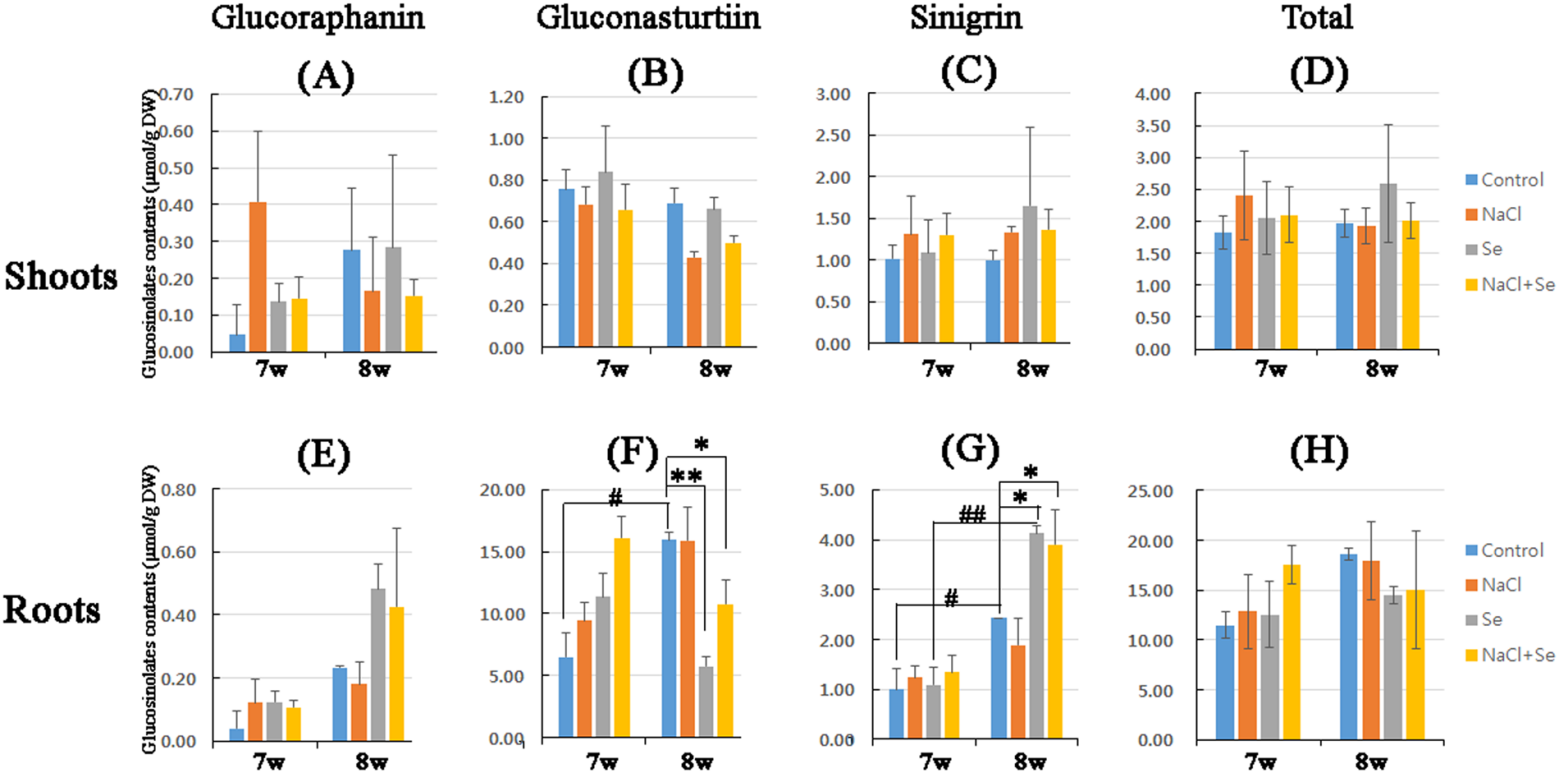
Analysis of levels of three glucosinolates (GLSs) in kale shoots and roots under conditions of stress. Plants grown in the plant factory were treated with control (blue), sodium chloride (NaCl, 80 mM; orange), selenite (Na_2_SeO_3_, 2 mg∙l^−1^; Se, grey), or a combination of both (NaCl + Se, yellow) for 6 weeks. Plants were harvested at week 7 or 8 and ground after freeze drying. The powder (0.2 g) was extracted using boiling 70% methanol and analysed for (**A** and **E**) glucoraphanin, (**B** and **F**) gluconasturtiin (GSN), (**C** and **G**) sinigrin, or (**D** and **H**) total GLS contents using high-performance liquid chromatography (HPLC). Levels were detected in three separate samples. Statistical comparisons of significance are shown in adjoining lines. *^,#^P < 0.05, **P < 0.005, and ^##^P < 0.005.

To assess the effects of NaCl or Na_2_SeO_3_ on kale metobolites, plants grown in the factory were treated with either one or both substances during week 6 and were subsequently harvested at week 7 or 8. The levels of the three GLSs (glucoraphanin, gluconasturtiin, and sinigrin) and ITCs (SFN, PEITC, and AITC) in the freeze-dried kale shoots or roots were measured using high-performance liquid chromatography (HPLC). The GLS content was higher in the roots than it was in the shoots of the kale plants (Fig. 4D and H). Treatment with NaCl, Na_2_SeO_3_, or both increased the GLS content relative to that of the control during week 7 and 8. Gluconasturtiin (Fig. 4B and F) and sinigrin (Fig. 4C and G) levels were higher in the roots than they were in the shoots, whereas no difference in glucoraphanin level was observed between the two plant tissues (Fig. 4A and E). There also was no statistically significant difference in the glucoraphanin content between the control and treated kale plant roots at week 7 and 8 (Fig. 4E). However, treatment with NaCl, Na_2_SeO_3_, or both caused significant changes in the gluconasturtiin and sinigrin content of kale roots at week 7 and 8.

The gluconasturtiin and sinigrin contents were higher at week 8 than they were at week 7 in the control plants, but treatment with Na_2_SeO_3_ alone or in combination with NaCl increased the sinigrin concentration at week 8 compared with that at week 7. In contrast, the gluconasturtiin content of the kale roots was reduced by Na_2_SeO_3_ treatment with or without NaCl, whereas NaCl alone had no effect. A significant increase in the total GLSs (Fig. 4H) was induced by co-treatment with NaCl and Na_2_SeO_3_ compared with levels in the untreated control at week 7. Levels were also higher in the control at week 8 than at week 7. These results demonstrate that a 2-week exposure to Na_2_SeO_3_ alone or combined with NaCl affected the gluconasturtiin and sinigrin concentrations in kale roots but not shoots.

**Figure 5:**
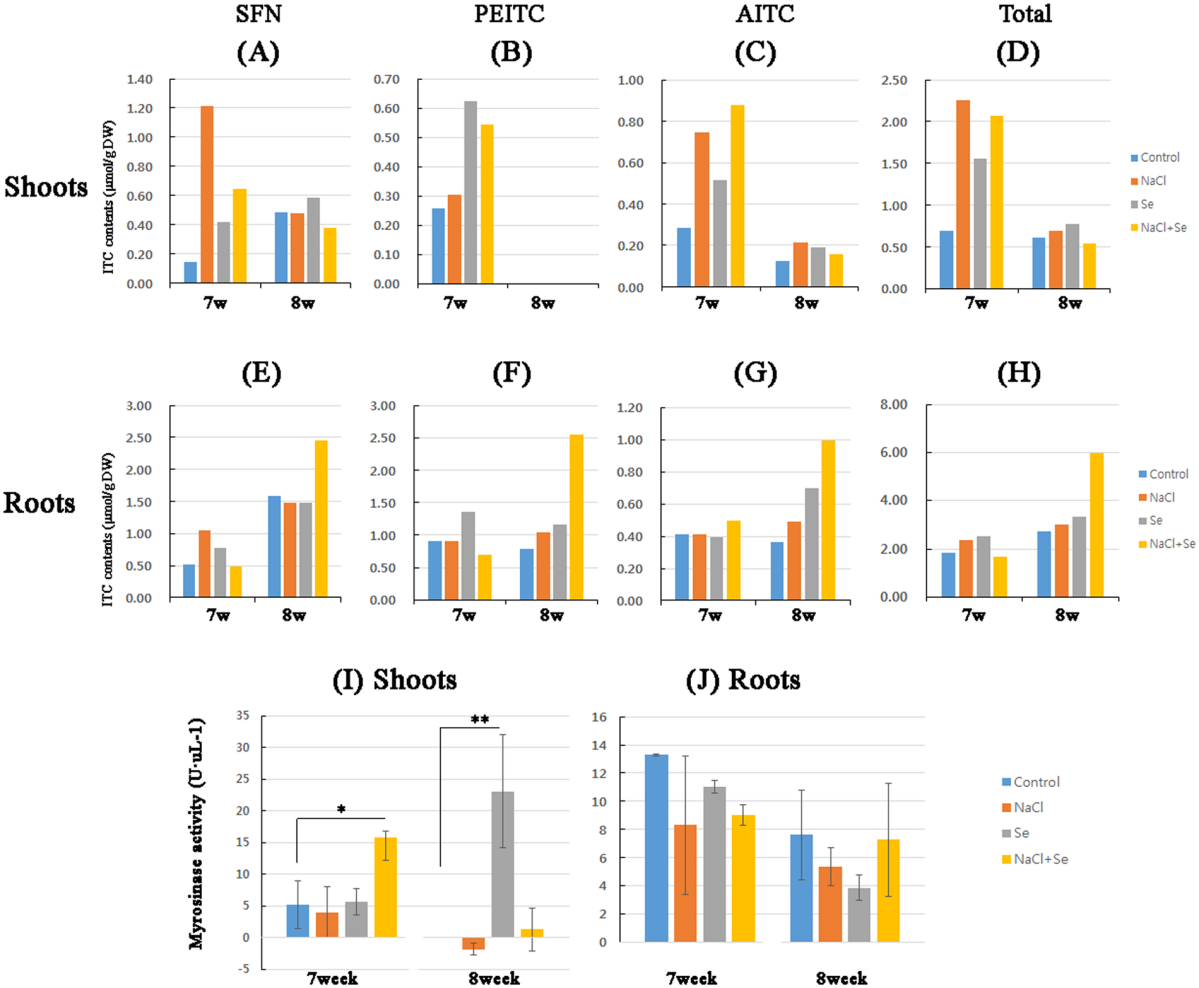
Analysis of levels of three isothiocyanates (ITCs) and myrosinase activity in kale shoots and roots. Kale grown in the plant factory were exposed to control (blue), sodium chloride (NaCl, 80 mM; orange), selenite (Na_2_SeO_3_, 2 mg∙l^−1^; Se, grey), or a combination of both (NaCl + Se, yellow) for 6 weeks. Plants were freeze-dried after harvesting at week 7 or 8. The powder (0.2 g) was extracted and analysed for (**A** and **E**) sulphoraphane (SFN), (**B** and **F**) phenylethyl ITC (PEITC), (**C** and **G**) allyl ITC (AITC), and (**D** and **H**) total ITC contents using high-performance liquid chromatography (HPLC). Myrosinase activity in (**I**) shoots and (**J**) roots was determined based on glucose concentration. The levels were detected in three separate samples. Statistical comparisons of significance are shown in adjoining lines. *P < 0.05 and **P < 0.005.

We analysed the ITC concentrations in kale, but the standard deviation could not be calculated for the data because the ITC content of the plants was typically detected with a wide error^17,18^. NaCl and Na_2_SeO_3_ alone or in combination increased the ITC levels in the shoots at week 7 compared with the levels at week 8 (Fig. 5D). However, the opposite trend was observed in the roots, which showed higher levels at the later time point (Fig. 5H). NaCl and Na_2_SeO_3_ also increased the SFN, PEITC, and AITC levels in the roots at week 8. Thus, kale shoots and roots produced more ITCs in the presence of NaCl, Na_2_SeO_3_, or both at the earlier and later time points (Fig. 5A–C and E–G).

### Myrosinase activity in kale is affected by NaCl and Na_2_SeO_3_ treatment

The enhancement of ITC production by GLS hydrolysis is important for myrosinase activity in kale^19^. To determine whether myrosinase activity is affected by NaCl, Na_2_SeO_3_, or both, we measured the glucose concentration in kale extract. The myrosinase activity of kale shoots at week 7 was increased by co-treatment with NaCl and Na_2_SeO_3_, whereas either agent alone had no effects (Fig. 5I). The myrosinase activity of kale shoots at week 8 was increased in the presence of Na_2_SeO_3_ (Fig. 5I), which had no significant effect on kale roots. These data suggest that myrosinase activity in the shoots but not the roots was altered by NaCl and Na_2_SeO_3_ at week 7 and by Na_2_SeO_3_ at week 8.

### Effect of kale extract of plants treated with NaCl, Na_2_SeO_3_, or both on Nrf2 expression

**Figure 6:**
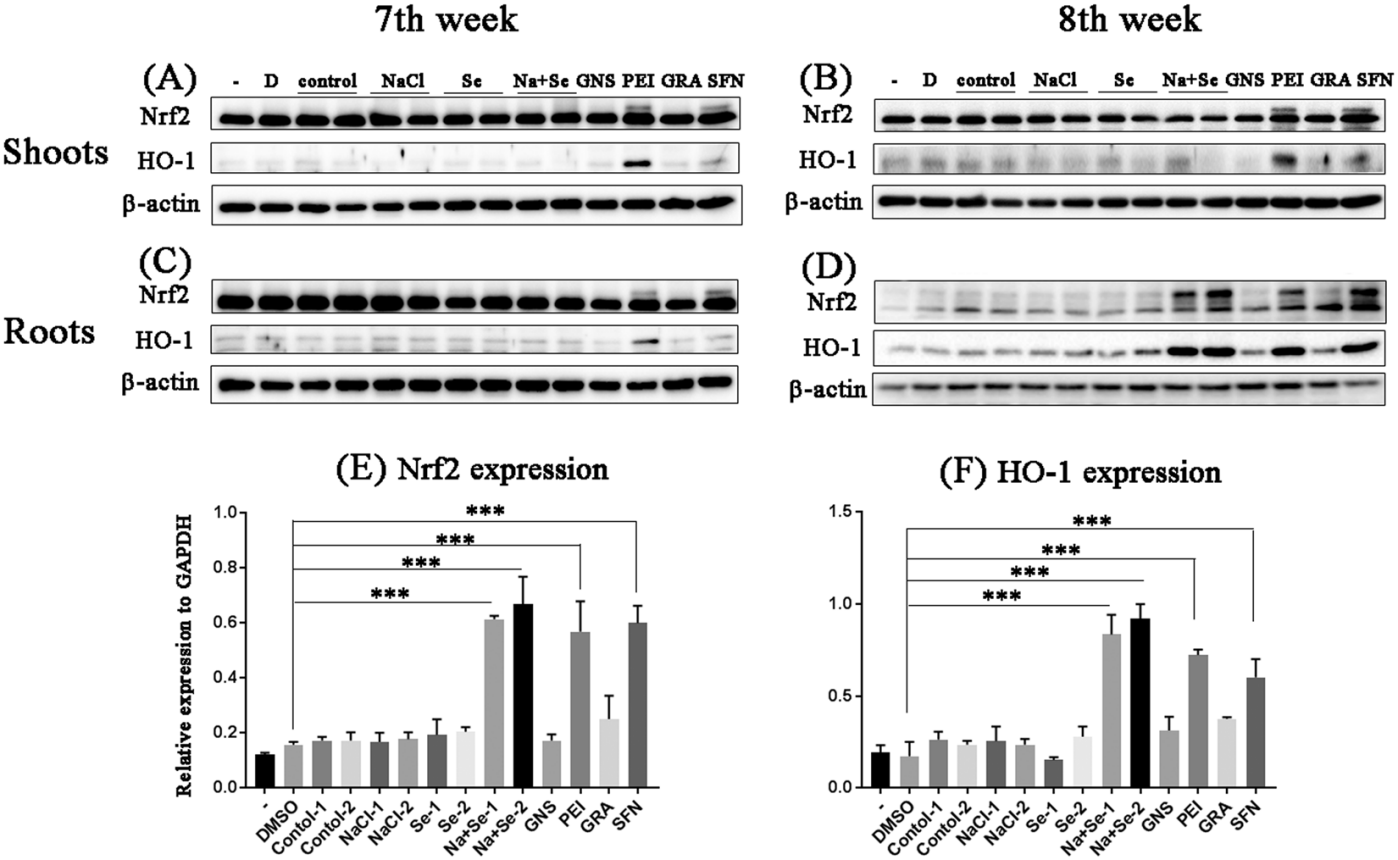
Extracts of kale roots treated with sodium chloride (NaCl) and selenite (Na_2_SeO_3_) increased Nrf2 expression at week 8. Kale leaves or roots were untreated (control) or treated with NaCl (80 mM), Na_2_SeO_3_ (2 mg∙l^–1^), or both, freeze-dried, and extracted with 70% ethanol. HepG2 cells were exposed to (**A**–**D**) extracts or positive controls (glucoraphanin [GRA], gluconasturtiin [gluconasturtiin], sulphoraphane [SFN], and phenylethyl isothiocyanate [PEITC]) for 6 h. Protein was extracted, and western blotting was carried out using antibodies against Nrf2 and HO-1 antibody, with β-actin as a loading control. Three separate samples were analysed. Statistical comparisons of significance are shown in (**E**) and (**F**). ***P < 0.0005.

The analysis of the GLS and ITC contents suggests that the detoxification pathway was activated by kale root extract. To investigate this possibility, HepG2 cells were exposed to the extract of kale plants treated with NaCl, Na_2_SeO_3_, or both for 6 h while gluconasturtiin, phenylethyl ITC, glucoraphanin (GRA), and SFN served as positive controls. Nrf2 and haeme oxygenase (HO)-1 expression was evaluated using western blotting. At week 7, no extract altered Nrf2 or HO-1 expression^20^ (Fig. 6A and C). However, the root extract of plants treated with both NaCl and Na_2_SeO_3_ for 8 weeks increased the Nrf2 and HO-1 levels (Fig. 6D). Shoot extracts at the same time point, and conditions had no effect on the expression of these proteins.

**Figure 7:**
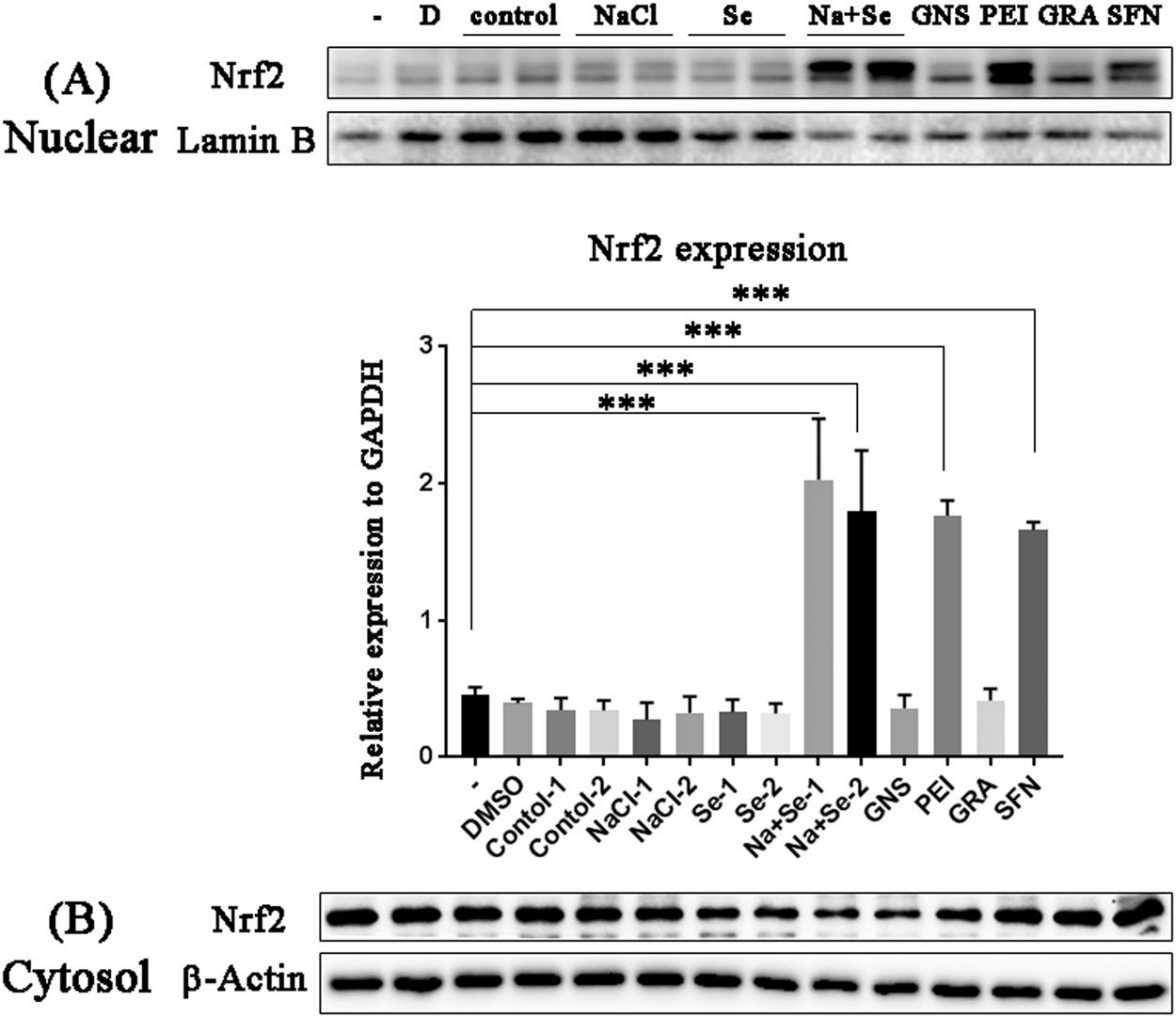
Nrf2 translocation is induced by kale roots treated with sodium chloride (NaCl) and selenite (Na_2_SeO_3_). HepG2 cells were exposed to extracts of kale roots that were untreated (control) or treated with NaCl (80 mM), Na_2_SeO_3_ (2 mg∙l^−1^) for 8 weeks. Glucoraphanin (GRA), gluconasturtiin (gluconasturtiin), sulphoraphane (SFN), and phenylethyl isothiocyanate (PEITC) were used as positive controls. Proteins were separated into (**A**) nuclear and (**B**) cytosolic fractions. Nrf2 was detected using western blotting with β-actin or lamin B as loading controls for cytosolic and nuclear fractions, respectively. Three separate samples were analysed. Statistical comparisons of significance are shown in (**A**). ***P < 0.0005.

Nrf2 subcellular localisation is important for its function^9^. To determine whether Nrf2 nuclear translocation is induced by NaCl or Na_2_SeO_3_ treatment, cell extracts were separated into the cytosolic and nuclear fractions. Extracts of kale root co-treated with NaCl and Na_2_SeO_3_ for 8 weeks showed Nrf2 nuclear accumulation (Fig. 7A), whereas no effects were observed with the other extracts. This confirmed that the extract of kale treated with NaCl and Na_2_SeO_3_ for 8 weeks induced the nuclear translocation of Nrf2 and induced Nrf2 and HO-1 expression in HepG2 cells as effectively as PEITC and SFN.

## Discussion

This study investigated the possibility of altering GLS and ITC production in kale grown in a plant factory to yield plants with a higher therapeutic value. Kale plants cultivated in the factory showed higher SFN concentrations than those grown in an open field did. The concentration of bioactive compounds can be maintained or even enhanced under artificial growing environments to a degree that is impossible in natural settings^21^. The fresh and dry weight of the kale shoots and roots were not significantly decreased by NaCl and Na_2_SeO_3_ treatment. These results imply that shoot and root growth of kale was influenced neither by NaCl nor Na_2_SeO_3_. A study reported that the growth of young kale plants was stimulated by increasing the Na_2_SeO_3_ concentration to up to 30 μg/mL, but was inhibited at higher concentrations^22^. At concentrations closer to 0 mg/L of selenium within the range of 3.2 mg/L, higher total GLS was observed, in young *Arabidopsis thaliana* and *Brassica Oleracea*^23^. Selenium treatment in the range of 0–4 mg/L, increased the shoot fresh weight dose-dependently. The highest root fresh weight and mineral contents were previously observed in plants treated with 2 mg/L selenium^24^. Based on these previous studies, we decided to use a concentration of 2 mg/L Na_2_SeO_3_ in our present study.

A water-limited environment induces the production of aliphatic GLSs^25,26^. In fact, salt stress (low-water conditions) has been used as a strategy to increase GLS levels in radishes^27^ and broccoli^28^ sprouts, suggesting that salt-rich conditions favour GLS production^29^. One study found that treatment of kale with sinigrin, an aliphatic GLS, under salt stress increased aquaporin expression in the roots, suggesting that sinigrin functions as an osmotic regulator for water conservation^30^. In contrast to previous reports, our results showed that salt stress had no effect on sinigrin concentration in the roots or shoots of kale, suggesting the *Brassica* spp. exhibit variable salt tolerance. The glucoraphanin level in the roots and shoots was also unaffected by the addition of salt, which is consistent with the finding that glucoraphanin is not influenced by salinity^28^. The level of gluconasturtiin, an aromatic GLS, was decreased in the presence of 100 mM salt^31^ due to reduced activity of phenylalanine ammonia lyase^32^, which catalyses the transformation of phenylalanine to aromatic GLSs^33^. Thus, the relatively low salt concentration (80 mM) used in this study did not alter gluconasturtiin levels in the shoots or roots. In addition, it has been demonstrated that GLS level was increased with age in the leaves from kale plants grown for up to 18 weeks^34^. Although the duration of our experiment was relatively short, the levels of gluconasturtiin and sinigrin increased between week 7 and 8 in control plants, which was observed in the roots and not the leaves. Our findings suggest that kale plants have a wide range of salt tolerance, although this has not been extensively investigated^35^.

Na_2_SeO_3_ is chemically similar to and competes with sulphur to interfere with sulphur assimilation in plants^36^. Na_2_SeO_3_ supplementation increased sulphur uptake but decreased glucoraphanin and sinigrin levels in the aerial parts of *B*. *oleracea*^37,38^. In contrast, treatment of *B*. *oleracea* L. var. italic with Na_2_SeO_3_ had no effect on glucoraphanin concentration^39^. We observed that the glucoraphanin level was unaffected while sinigrin concentration was increased by Na_2_SeO_3_ in the roots, which was not altered by salt stress. In addition, Na_2_SeO_3_ caused a decrease in gluconasturtiin content that was partly mitigated by co-administration of salt. These results indicate that Na_2_SeO_3_ has different effects on the metabolism of individual GLSs in kale roots, which is regulated by distinct mechanisms^40,41^. An earlier study reported that the metabolism of GLSs was dependent on the R functional group^38^. Specifically, glucoraphanin and sinigrin production was regulated by alleles at the *Gsl-oxid* and *Gsl-alk* loci, respectively. Moreover, the GLS and ITC profiles of kale roots and shoots were differentially affected by NaCl and Na_2_SeO_3_ treatment, which may be attributed to varying transcriptional responses to NaCl stress between roots and aerial parts of the plant, which was demonstrated by RNA sequencing in *Brassica napus*^42^. Thus, NaCl and Na_2_SeO_3_ alone or in combination could alter the transcription or enzyme responsible for sinigrin and gluconasturtiin metabolism, leading to their up- and down-regulation, respectively, in kale root.

Based on the above results, we hypothesized that the increase in GLS content was associated with a similar increase in ITC levels and, therefore, we also analysed the myrosinase activity to clarify the mechanistic basis for these changes. In kale shoots, the levels of three ITCs were dependent on myrosinase activity. Specifically, the observed decrease in GLS concentration from week 7 to 8 was accompanied by a reduction in myrosinase activity. However, the reason for the increase in myrosinase activity with Na_2_SeO_3_ treatment remains to be clarified. The enzymatic activity of the kale roots was less sensitive to treatment with NaCl, Na_2_SeO_3_, or both than the shoots were. The co-treatment markedly increased the levels of the three ITCs in kale roots, whereas the myrosinase activity remained unchanged. These results suggest that the ITC concentration was unaltered as long as myrosinase activity remained the same. Moreover, it is possible that there is an alternative pathway that is responsible for ITC production^43^. Changes in myrosinase activity in response to treatment with NaCl or Na_2_SeO_3_ have been shown to vary across plant species even under the same treatment conditions^44^, for instance in germinating seeds of the *Brassica* family^45^, radish sprouts^27^, broccoli sprouts^14,28^, and mature broccoli^46^. We speculated that differences in sample preparation to preserve the enzyme—e.g. freezing only, freeze-drying, or no freezing—contribute to variations in enzyme activity, which warrants further study. Interestingly, the translocation of Na_2_SeO_3_ from the roots to shoots was dependent on the form of Na_2_SeO_3_ supplied. In Indian mustard, only 10% of the Na_2_SeO_3_ uptake was transported from the roots to shoots^47^. In the bean plant, Na_2_SeO_3_ accumulated in the roots and only a small fraction was transported to the shoot^48^. Since Na_2_SeO_3_ is rapidly converted to organic forms such as SeMet that are retained in the roots, very little localizes to the shoots^49^. Therefore, co-treatment with NaCl and Na_2_SeO_3_ may create a more stressful condition for kale, which could affect the GLS and ITC generation.

Most strikingly, we observed that SFN, PEITC, and AITC levels were increased in kale roots by the co-treatment at week 8, which was associated with enhanced chemopreventive effects on HepG2 cell lines. It has been well established that SFN^50^ and PEITC^51^ regulate Nrf2 via phosphorylation of extracellular signal-regulated kinase 1/2 and c-Jun N-terminal kinase 1 and not phosphoinositide 3-kinase/Akt signalling. Although the molecular target of AITC is unknown, our data indicate that these three ITCs are mainly responsible for the observed increases in Nrf2 and HO-1 expression.

In conclusion, this study provides evidence that the health benefits and chemopreventive effects of kale roots can be enhanced by cultivation in a plant factory system where the environmental conditions are manipulated by NaCl or Na_2_SeO_3_ supplementation to increase GLS and ITC levels.

## Methods

### Plant material and growth conditions

The experiment was carried out using commercial kale plants (*B*. *oleracea* L. var. sabellica, Asia Seed Co., Seoul, Korea) at SMART u-FARM in KIST (Gangneung, Korea). Kale seeds were sown in moist rockwool cubes (W × L × H, 25 × 25 × 40 mm, respectively, Grodan Co., Roermond, The Netherlands) and covered with a transparent acrylic plate under fluorescent lamps [6500 K, photosynthetic photon flux density (PPFD) = 200 ± 11 μmol^−2^s^−1^, 25 cm from the light source, TL5 14 W/865, Philips, Seoul, Korea) on a 14:10-h light/dark cycle at 26–18 °C and 50–90% relative humidity under closed cultivation conditions. Plants were transplanted to a deep-flow technique hydroponic system 8 days after sowing, and then to a 12-section nutrient film technique (NFT) hydroponic system on day 22. The hydroponic system provided a nutrient solution containing 7.0, 3.0, 4.5, 2.5, 1.0, and 1.0 me∙l^−1^ of P, K, Ca, Mg, S, and N, respectively as well as micronutrients. The solution was replaced weekly.

Plants were grown under fluorescent lamps (6500 K, PPFDs = 200 ± 15 μmol∙m^−2^s^−1^, 30 cm from light source, FL20EX-D 18 W, Shin Kwang, Mungyeong, Korea) on a 14:10-h light/dark cycle at 26–18 °C and 50–80% relative humidity, with 800 ± 35 ppm CO_2_ during the light period and an electrical conductivity (EC) value of 1.2 dS∙m^−1^ (Fig. 1). At 6 weeks after sowing, the three sections of the NFT hydroponic system were exposed to the vehicle, 2 mg∙l^−1^ Na_2_SeO_3_, 80 mM NaCl, or a combination of Na_2_SeO_3_ and NaCl for 1 or 2 weeks (Supplementary Fig. S1). At week 7 or 8 (i.e. 1 or 2 weeks of treatment, respectively), the shoots and roots were separately harvested. Prior to treatment, the nutrient solution concentration was maintained under EC 1.5 dS/m; after treatment, the concentration was maintained for 2 weeks at EC 1.17 ± 0.18 (control), EC 10.8 ± 0.75 (NaCl), EC 1.47 ± 0.58 (Na_2_SeO_3_), and EC 10.5 ± 0.86 (NaCl + Na_2_SeO_3_, Fig. S2). For comparison, identical kale plants were transplanted to an open field 8 days after sowing and were cultivated for the same period as the plants in the factory. A nutrient solution containing the same ratios of ions described above with an EC 0.5 was supplied weekly.

### Preparation of plant material

Harvested kale plants were separated into aerial parts (leaves and stem) and the roots. Tissue samples were freeze-dried for 4 days, and 1 g of each sample was ground for glucosinolate analysis while 500 g of each sample was extracted with 70% ethanol for 14 days and dried for western blot analysis.

### Determination of GLS content in kale samples

Sample preparation and GLS analysis were performed as previously described^52^. Briefly, freeze-dried kale powder (0.2 g) was extracted with 2 mL boiling 70% methanol for 10 min. After cooling on ice, 0.5 mL benzyl-GLS (1 mM) was added as an internal standard, followed by mixing and centrifugation at 3,000 rpm for 15 min at 4 °C. The supernatant was saved and the pellet was re-extracted with 2 ml of 70% methanol at 95 °C for 10 min and the two extracts were combined. The crude GLS extract (1 mL) of each pooled extract was transferred into a 2-mL microcentrifuge tube. The protein was precipitated with 0.15 mL of a 1:1 mixture of 1 M lead acetate and 1 M barium acetate. After centrifugation at 12,000 rpm for 5 min, samples were loaded onto a column containing diethyl-aminoethyl (DEAE) Sephadex A-25 anion exchange resin, which was pre-activated with 0.1 M sodium acetate. Desulphation was carried out by adding 500 μL purified aryl sulphatase (*Helix pomatia* Type H-1, Sigma-Aldrich, St. Louis, MO, USA).

The column was capped and allowed to stand at room temperature for 18 h, and desulpho-GLS was eluted with 3 mL distilled water and filtered through a 0.2-μm polyvinylidene difluoride (PVDF) syringe filter. A 10-μL aliquot of each sample was injected into a 1200 series HPLC system (Agilent Technologies, Santa Clara, CA, USA) using a YMC-Pack Pro C8 column (4.6 × 150 mm, 3 μm; YMC Mechanical, Meridian, ID, USA), and the absorbance spectrum was measured at 227 nm using a photodiode array detector. The mobile phase was 30% acetonitrile and water, and the flow rate was maintained at 0.7 mL∙min^−1^. The gradient program was as follows: a linear step from 0% to 100% of solvent A within 20 min, followed by constant conditions for up to 30 min. GS was quantified as previously described^53^.

### Analysis of ITC concentration in kale

Freeze-dried kale powder (75 mg) was vortexed with 1.5 mL distilled water and incubated for 24 h at room temperature in the dark. Samples were centrifuged at 15,000 rpm for 10 min, and the supernatant was mixed with benzyl ITC and methylene chloride. After centrifugation, the bottom layer was removed and incubated with derivatisation reagent containing triethylamine and β-mercaptoethanol for 1 h at room temperature. Samples were vacuum-dried in an oven for 1 h and dissolved in acetonitrile and water (1:1 ratio). ITCs were quantified using HPLC with an Atlantis T3 column (4.6 × 150 mm, 5 μm; Waters, Milford, MA, USA).

### Determination of myrosinase activity based on glucose level

Freeze-dried kale powder (0.1 mg) was extracted in distilled water. Myrosinase activity was detected using a glucose assay kit (Sigma-Aldrich) according to the manufacturer’s protocol.

### Cell culture

HepG2 human hepatocellular carcinoma cells (Korean Cell Line Bank, Seoul, Korea) were used to assess the antioxidant protein expression. Cells were grown in minimum essential medium (Welgene, Dea-gu, Korea) supplemented with 10% foetal bovine serum (Welgene), 100 U∙mL^−1^ penicillin, and 100 μg∙mL^−1^ streptomycin (Hyclone, Logan, UT, USA) at 37 °C in a humidified atmosphere of 5% CO_2_.

### Nuclear and cytosolic fractionation

HepG2 cells were treated with 20 μL of the plant tissue extracts (20 mg∙ml^−1^) or a positive control (5 μM; GRA, gluconasturtiin, SFN, and PEITC) for 6 h. A nuclear extraction kit (Cayman Chemical, Ann Arbor, MI, USA) was used for nuclear fractionation according to the manufacturer’s protocol. The target protein expression was evaluated using western blotting.

### Western blotting

The protein concentration in HepG2 cell lysates (8 μg) was quantified using the Bradford assay (Bio-Rad, Hercules, CA, USA), and proteins were separated by 8% Tris-glycine sodium dodecyl sulphate–polyacrylamide gel electrophoresis and transferred to polyvinylidene fluoride (PVDF) a membrane (Millipore, Billerica, MA, USA) that was blocked with 5% bovine serum albumin for 1.5 h at room temperature. The full length blot was cropped before incubation with individual antibody. The each membrane was then incubated overnight at 4 °C with antibodies against Nrf2 (Abcam, Cambridge, MA, USA; 1:1000); HO-1, lamin B (both from Santa Cruz Biotechnology, Santa Cruz, CA, USA; 1:1000), β-Actin (Santa Cruz Biotechnology; 1: 10,000) served as the loading control. Primary antibodies were detected using horseradish peroxidase-conjugated secondary antibodies (Santa Cruz Biotechnology; 1:5000), and immunoreactivity was visualized using an enhanced chemiluminescence system (Thermo Fisher Scientific) along with a ChemiDoc XRS + (Bio-Rad). The exposure time of membrane was between 0.5 to 2 second and we did not conduct additional process. The relative amount of protein was divided by the corresponding amount of β-Actin using Image Lab v.5.2.1 software (Bio-Rad).

### Statistical analysis

The data were subjected to a two-way analysis of variance using the statistical package for the social sciences (SPSS) v.18.0 software (SPSS Inc., Chicago, IL, USA). The variation was related to the main treatments (NaCl and Na_2_SeO_3_) and their interaction. The means ± standard deviations (SD) was calculated and when the F-ratio was significant, the least significant difference (LSD) was evaluated using Duncan’s test. Differences were considered significant at P < 0.05.

## Electronic supplementary material

Supplementary information
