# Supplementary material for: Exposure of kale root to NaCl and Na2SeO3 increases isothiocyanate levels and Nrf2 signalling without reducing plant root growth
Source: Sci Rep. 2018 Mar 5;8:3999. doi: 10.1038/s41598-018-22411-9 (PMC5838157; doi:10.1038/s41598-018-22411-9)
Supplement: Supplementary file 1 — Supplementary information [file 41598_2018_22411_MOESM1_ESM.pdf]

Exposure of kale root to NaCl and Na_2SeO_3 increases isothiocyanate levels and Nrf2 signalling without reducing plant root growth

Sun Young Kim[1#], Jai-Eok Park[1#], Eun Ok Kim[1], Sue Ji Lim[1], Eui Jeong Nam[1], Ji Ho Yun[1, 2], GyHye Yoo[1], Sang-Rok Oh[1], Hyoung Seok Kim[1], and Chu Won Nho[1,*]

	Week	1 W							2 W							4 W							6 W							7 W							8 W						
	No. of date	1	2	3	4	5	6	7	8	9	10	11	12	13	14	22	23	24	25	26	27	28	36	37	38	39	40	41	42	43	44	45	46	47	48	49	50	51	52	53	54	55	56
Stage of culture	Seeding	○																																									
	Garmination			○																																							
	Transplant								○																																		
	Planting															○																											
	Stress treatment																												○														
	Harvest																																			○							○

Growth environment	EC		1.2 $dS\cdot m^{-1}$	CON: EC 1.2 dS·m-1
				EC 1.2 + selenite (0.05 mg·L^{-1} Na_2SeO_3)
				EC 1.2 + seline (80 mM NaCl)
				EC 1.2 + selinite + seline
	Temperature	26 - 18 ℃	24 - 18 ℃	
	RH	50 - 90 %	50 - 80 %	
	CO_2	non-supply	800 ppm for day time, non-supply for night time	
	Light period	14h day and 10h night		

Supplementary Figure S1. Plant materials cultivation and nutrient solution treatment scheme.
